# An evaluation on the effect of the copayment waiver policy for Korean hospitalized children under the age of six

**DOI:** 10.1186/s12913-015-0836-x

**Published:** 2015-04-20

**Authors:** Sook Young Kwak, Seok-Jun Yoon, In-Hwan Oh, Young-eun Kim

**Affiliations:** Bureau of Welfare Administration Support, Ministry of Health and Welfare, Sejong, South Korea; Department of Preventive Medicine, College of Medicine, Korea University, Seoul, South Korea; Department of Preventive Medicine, College of Medicine, Kyung Hee University, Seoul, South Korea; Division for Healthcare Technology Assessment Research, National Evidence-based healthcare Collaborating Agency (NECA), Seoul, South Korea

**Keywords:** Copayment, Copayment waiver policy, Medical utilization, Difference in difference, Korea

## Abstract

**Background:**

In January 2006, the Korean government implemented a copayment waiver policy for hospitalized children under the age of 6 years to reduce the economic burden on patients. This policy was implemented from 2006 to 2007 in Korea and involved hospitalized children under the age of 6 years. The goal of this study is to evaluate the effect of the copayment waiver policy on health insurance beneficiaries.

**Methods:**

The change in medical service utilization before and after the policy implementation was analyzed using data from the national health insurance corporation (NHIC) and compared with medical aid beneficiaries who were already exempt from copayment. The “difference in difference” method was applied to determine the net effect of the copayment waiver policy.

**Results:**

The net effect of policy implementation on NHIC beneficiaries was unclear by the “difference in difference” method because the number of inpatient days and hospital expenditure after policy implementation showed opposite results. The copayment waiver policy did not decrease the intensity of health care utilization when compared with the medical aid beneficiaries group. Among the NHIC beneficiaries, patients who utilized medical services for fatal disease and those with the low premiums group were more affected by the policy.

**Conclusions:**

The net effect of copayment waiver policy remains unclear. Therefore, further studies are needed to determine the effects of policies implemented to reduce the economic burden on patients, such as the herein-described copayment waiver policy.

## Background

Korea provides mandatory social health insurance from the National Health Insurance Corporation (NHIC) for its entire population, with exception of the poorest 3-5%, who are covered by medical aid [1]. The benefit coverage for NHIC beneficiaries and medical aid beneficiaries is identical. Korea’s national health insurance is financed by premiums from insured and government subsidies. In 2007, premiums occupied 83.4% of NHIC revenue and government subsidies occupied 14.1% [2]. For industrial workers which include government employee, the premium of national health insurance is proportional to wage and it is equally shared by the employer and employee. The contribution rate of industrial worker was 5.08% of wage in 2008. For self-employed, the premium is imposed by a formula based on gender, income and property to reflect actual income. Additionally, NHIC beneficiaries pay a copayment [3]. In case of outpatient care, copayment ranges from 30% of clinic visit to 60% of higher level general hospital. For the inpatients case, the copayment rate is 20%. For some diseases such as cancer and catastrophic event that copayment exceeds $2900 dollar, discount and ceiling for copayment was applied. But this is only applied small portion of total hospitalization case [2].

Medical aid is funded by the central and local governments, allowing the beneficiaries of medical aid to pay a relatively lower copayment [4]. NHIC remunerated providers and is refunded by governments [5]. Beneficiaries of medical aid who are less than 18 years old do not have a copayment. However, elective procedures such as cosmetic enhancement and surgery to end snoring are excluded from the NHIC and medical aid benefit coverage [3].

In January 2006, the Korean government implemented a copayment waiver policy for hospitalized children under the age of 6 years who are covered by NHIC with the aim of minimizing the economic burden on patients. The copayment for hospitalized children under the age of 6 years was set at 0%; however, at the end of 2007, the Korean government decided to stop the policy because of the huge financial expenditure, and increased the copayment rate for these children to 10% of the total cost.

Many studies on health insurance policies and health care utilization have been performed. For example, a study on the expansion of the State Children’s Health Insurance Program (SCHIP) in the United States, found that SCHIP enrollment improved access to health care and increased the quality of care, especially for children in low income families [6,7]. In Korea, low income patients and users of clinics are sensitive to the copayment rate [8]. Additionally, the separation of drug prescriptions and dispersal in Korea reportedly increased the public share of total health expenditure [9]. However, the effect of the copayment waiver policy on children under the age of 6 years has not been evaluated. In addition, the studies on copayment waiver are scarce as well.

The goal of this research is to evaluate the effect of the copayment wavier policy during its 2 years of implementation. Because the beneficiaries of medical aid under the age of 6 years were already exempt from copayment, the copayment waiver policy included only the beneficiaries of the NHIC. Therefore, this study compared the differences between medical aid beneficiaries and NHIC beneficiaries and aimed to reveal the net effect of the copayment waiver policy on children. The NHIC beneficiaries were divided into subgroups, and the authors attempted to determine the differences in effect according to disease category and income level, as measured by the NHIC premium level. This study was conducted with consideration of the interactions of the policy and its variables.

## Methods

### Data collection and classification

First, data on the inpatient NHIC beneficiaries from 0 to 5 years of age were collected. Data on patients hospitalized before (1 August 2004 to 30 November 2005) and after (1 February 2006 to 31 May 2007) implementation of the policy was collected to assess the net effect of the policy. The NHIC beneficiaries were subgrouped as two groups −1) industrial worker and their dependents and 2) self-employed and their dependent. Data on inpatient care of medical aid beneficiaries hospitalized from 0 to 5 years were also collected during the same period to compare the net effect of the policy.

Diseases were categorized as either fatal or non-fatal to analyse the effects of each policy. Data from the National Statistical Office in 2006 were used to classify fatal diseases [10]. Forty-eight leading causes of death for children aged 0 to 5 years were selected in order of frequency and were considered to be fatal diseases

The premiums of the beneficiaries of NHIC were used as the proxy indicators of income level to assess the effects on NHIC beneficiaries according to income group. In Korea’s NIH system, the premium level depends on the income level of the beneficiary. Additionally, the households were divided into five groups of equal numbers of subjects based on the premium level [3]. The effects of the policy were measured by medical utilization. The number of inpatient days per case, and the hospital expenditure per case were used as the indictor of medical utilization. The patients’ copayments and the costs covered by the NHIC were included in the hospital expenditure. The medical utilization of NHIC beneficiaries were compared with those of medical aids group.

### Data analysis

The “difference in difference is used to reduce the probabilities of time-invariant omitted variables and time trends by comparing results before the intervention and after interruption and by comparing the intervention group with the control group. The differences in the intervention group before and after the intervention should be compared with those of the control group before and after the intervention [11].

A regression equation was used to estimate the net effect of the policy. In the regression equation, individual characteristics—including age, sex, premium level, and disease characteristic (fatal or non-fatal)—were used as independent variables. Intensity indices—such as the number of inpatient days per case and hospital expenditure per case—were used as dependent variables. The equation used is shown below.

$$ {\mathrm{S}}_i: = \upalpha +{\upbeta}_1\mathrm{X}+{\upbeta}_2\mathrm{C}+{\upbeta}_3\mathrm{D}+{\upbeta}_4\mathrm{C}*\mathrm{D}+\mathrm{E} $$

S_*i*_: the number of inpatient days per case, hospital expenditure per case,logarithmic transformation

X: independent variables (age, sex, level of premium, type of medical institute, fatality of the disease)

C: group variable (0 = medical aid group, 1 = national health insurance group)

D: time variable (0 = copayment existence, before Nov.30. 2005; 1 = after Feb.1, 2006, after the policy)

β_4_: “difference in difference” estimator, the net effect of the copayment waiver policy

Indices of the intensity of medical use (*S*_*i*_), such as the number of inpatient days per case and the hospital expenditure per case were analyzed after logarithmic transformation, because the data for this research were not normally distributed. In this equation, β_4_: is the estimator of “difference in difference.” The degree of medical use between the NHIC beneficiaries group and the medical aid beneficiaries group was compared using the “difference in difference” method.

Additionally, the effects of the policy among NHIC beneficiaries according to disease and premium level were analyzed with consideration of the interaction of the policy and the stated variables. The patients with fatal and non-fatal diseases were compared to create a model with which to determine the effect of disease on healthcare use. Patients with non-fatal diseases comprised one reference group, and patients with the highest premiums (upper 20%) comprised another reference group to determine the effect of premium level on policy use. The Korean Won was converted to US dollars according to the 2007 average exchange rate of 1 USD to 929.26 Won [12].

In this study, the data of NHIC which did not have personal identifier were used, and the use of data was approved by NHIC. This survey confirmed to the Declaration of Helsinki as well as to local legislation. Statistical analysis was performed using SAS version 9.1 (SAS institute Inc. Cary, NC), and statistical significance level was set at 0.05.

## Results

Table 1 represents the changes in medical utilization between 2004.8.1-2005.11.30 and 2006.2.1-2007.5.31. In the NHIC beneficiaries group, the number of inpatient days per case decreased from 6.37 to 5.96, and the hospital expenditure per case decreased from $709.32 to $644.67. In the medical aid beneficiaries group, the number of inpatient days per case decreased from 13.48 to 10.53, and hospital expenditure per case decreased from $2067.94 to $1452.53. Among the NHIC beneficiaries group, the industrial worker group showed larger decrease.

**Table 1:** **Changes in medical utilization before and after** ^**a**^ **copayment waiver policy implementation**

	**Total patients <6 years of age**	**Total Inpatients <6 years of age**	**Average age**	**Inpatient days per case (n)**	**Hospital expenditure per case (USD)**
	National Health Insurance Group (Total)				
Before	429,666	535,129	1.86	6.37	709.32
After	699,254	891,278	1.28	5.96	644.67
Rate of Increase	62.7%	66.6%	-	−6.4%	−9.1%
	National Health Insurance Group(Industrial worker)				
Before	273,194	336,335	1.82	6.25	706.45
After	480,739	603,284	1.24	5.85	635.40
Rate of Increase	76.0%	79.4%	-	−6.5%	−10.0%
	National Health Insurance Group(Self-employed)				
Before	160251	198794	1.93	6.57	714.17
After	228610	287994	1.35	6.20	662.84
Rate of Increase	42.7%	44.9%	-	−5.6%	−7.2%
	Medical Aid Group				
Before	7860	11852	3.71	13.48	2067.94
After	16616	25469	2.94	10.53	1452.53
Rate of Increase	111.4%	114.9%	-	−21.9%	−29.8%

^a^Before (2004.8.1-2005.11.30) and after (2006.2.1-2007.5.31).

The overall trends of intensity in medical utilization among the NHIC beneficiaries are shown according to premium level in Table 2. The low premium group of NHIC beneficiaries showed the most significant decrease in the premium level after policy implementation (Table 2).

**Table 2:** **Change in rate by premium level after policy implementation**

**Premium Level**	**Inpatient days (%)**			**Hospital expenditure (%)**		
	**Total**	**Industrial worker**	**Self-employed**	**Total**	**Industrial worker**	**Self-employed**
Low	−9.0	−8.1	−11.6	−11.8	−10.2	−17.2
Low middle	−6.1	−6.6	−4.8	−8.8	−10.7	−5.4
Middle	−6.6	−6.7	−5.6	−10.4	−11.0	−9.0
High middle	−4.0	−3.9	−3.5	−6.6	−8.0	−3.7
High	−4.2	−3.7	−3.5	−8.2	−11.4	−4.0

The results of applying a logarithm to the intensity of medical utilization are shown in Table 3. The number of inpatient days per case exhibited a 0.101 interaction coefficient between the NHIC beneficiaries group and policy implementation. This value indicates that when analyzed using the indicated logarithm (number of inpatient days per case), the degree of decrease among the NHIC beneficiaries after policy implementation was smaller than that of the medical aid beneficiaries group. Regarding the hospital expenditure per case, the “difference in difference” estimator was −0.123, indicating that the degree of decrease in the hospital expenditure, among the NHIC beneficiaries as analysed by the chosen logarithm (medical expense per case), was greater than that among the medical aid beneficiaries.

**Table 3:** **Regression analysis results of comparison of national health insurance beneficiaries and medical aid beneficiaries after copayment waiver policy implementation**

**Variable**	**Inpatient days per case**			**Hospital expenditure per case**		
	**Total**	**Industrial worker**	**Self-employed**	**Total**	**Industrial worker**	**Self-employed**
Intercept	1.784^a^	1.791^a^	1.777^a^	11.874^a^	11.858^a^	11.929^a^
Sex	−0.004^a^	−0.003^a^	−0.008^a^	0.051^a^	0.053^a^	0.044^a^
Age	−0.019^a^	−0.022^a^	−0.018^a^	0.115^a^	0.118^a^	0.103^a^
Type of medical institute						
Tertiary hospital and General hospital	0.192^a^	0.191^a^	0.197^a^	1.096^a^	1.101^a^	1.085^a^
Hospital	0.080^a^	0.074^a^	0.100^a^	0.328^a^	0.309^a^	0.378^a^
Clinic	-	-	-	-	-	-
Etc. (Dental hospital, Health center, Oriental medical clinic)	−0.439^a^	−0.418^a^	−0.408^a^	−0.139^a^	−0.216^a^	0.028
Fatal disease	0.426^a^	0.430^a^	0.418^a^	0.557^a^	0.564^a^	0.536^a^
Group (Ref: medical aids group)	−0.428^a^	−0.452^a^	−0.393^a^	−0.177^a^	−0.178^a^	−0.188^a^
Time	−0.125^a^	−0.127^a^	−0.125^a^	0.035^a^	0.039^a^	0.023^a^
Group*time (difference in difference estimator)	0.101^a^	0.104^a^	0.104^a^	−0.123^a^	−0.133^a^	−0.097^a^
R^2^	0.102	0.108	0.105	0.354	0.364	0.329

^a^P-value < 0.05.

The interaction coefficient between the premium level or fatality and the policy is shown in Table 4. The interaction coefficient between fatal disease and policy implementation was 0.093, indicating a statistically significant interaction. This result indicates that use of the waiver policy affected NHIC beneficiaries with fatal and non-fatal diseases differently. The interaction coefficient for the hospital expenditure was 0.386, which suggests that the hospital expenditure per case was affected with fatal and non-fatal diseases differently by policy implementation.

**Table 4:** **Effects of copayment waiver policy implementation according to disease severity among National Health Insurance beneficiaries**

**Variable**	**Inpatient days per case**			**Hospital expenditure per case**		
	**Total**	**Industrial worker**	**Self-employed**	**Total**	**Industrial worker**	**Self-employed**
Intercept	1.296^a^	1.254^a^	1.339^a^	11.724^a^	11.696^a^	11.769^a^
Gender	−0.004^a^	−0.002	−0.007^a^	0.051^a^	0.053^a^	0.046^a^
Age	−0.018^a^	−0.020^a^	−0.017^a^	0.114^a^	0.118^a^	0.104^a^
Type of Medical Institute						
Tertiary hospital and General hospital	0.189^a^	0.188^a^	0.193^a^	1.078^a^	1.084^a^	1.066^a^
Hospital	0.075^a^	0.066^a^	0.090^a^	0.310^a^	0.288^a^	0.351^a^
Clinic	-	-	-	-	-	-
Etc. (Dental hospital, Health center, Oriental medical clinic)	−0.473^a^	−0.469^a^	−0.481^a^	−0.184^a^	−0.281^a^	−0.027
Fatal disease	0.370^a^	0.373^a^	0.365^a^	0.331^a^	0.329^a^	0.332^a^
Premium level						
Lower	0.114^a^	0.147^a^	0.111^a^	0.088^a^	0.102^a^	0.092^a^
Lower middle	0.109^a^	0.135^a^	0.088^a^	0.080^a^	0.096^a^	0.066^a^
Middle	0.078^a^	0.098^a^	0.066^a^	0.055^a^	0.067^a^	0.046^a^
High middle	0.040^a^	0.063^a^	0.030^a^	0.022^a^	0.033^a^	0.016^a^
High	-	-	-	-	-	-
Time	−0.032^a^	−0.015^a^	−0.037^a^	−0.163^a^	−0.167^a^	−0.148^a^
Fatal disease*time	0.093^a^	0.093^a^	0.092^a^	0.386^a^	0.393^a^	0.370^a^
Premium level*time						
Lower*time	−0.036^a^	−0.049^a^	−0.043^a^	−0.044^a^	−0.023^a^	−0.121^a^
Lower middle*time	−0.013^a^	−0.029^a^	−0.003	−0.028^a^	−0.035^a^	−0.021^a^
Middle*time	−0.014^a^	−0.029^a^	−0.003	−0.031^a^	−0.041^a^	−0.014
High middle*time	0.001	−0.013^a^	0.009	0.000	−0.006	0.013
High*time	-	-	-	-	-	-
R^2^	0.099	0.102	0.095	0.362	0.373	0.341

^a^P-value < 0.05.

Additionally, the result of Table 4 indicates that the degree of change in the highest premium level was smaller than that in the lower premium levels. Lower, lower middle and middle premium group’s inpatients days and hospital expenditure per case were greatly decreased in comparison to those of the high premium group after policy implementation.

## Discussion

This study evaluated the effect of the copayment waiver policy on hospitalized children under the age of 6 years. However, the effects of the copayment waiver policy on NHIC beneficiaries remain unclear after performing this analysis. The copayment waiver policy was associated with a smaller decrease in the number of inpatient days among NHIC beneficiaries than among medical aid beneficiaries. In contrast, NHIC beneficiaries showed a greater decrease in hospital expenditure than did medical aid beneficiaries after implementation of the copayment waiver policy. Therefore, the net effect of the copayment waiver policy was not clear.

The effects of copayment have been evaluated in various studies [13-15]. For example, implementation of copayment among Medicaid beneficiaries changed treatment pattern, though the total expenditure was not changed. The use and cost of pharmacy decreased but those of inpatients increased [16]. Also the increasing copayment for cancer patients resulted in the decrease of pharmacy cost and the increase of emergency room visit [17]. In case of emergency room visit, imposing copayment did not change the use of emergency room visit or inpatients care [18]. For ambulatory care, the implementation of copayment resulted in a reduction in the demand for ambulatory care, especially in low-economic-status areas [19]. But the effect of cost discount policy for the elderly is evaluated as limiting in Korea [20]. These data generally focused on outpatient care and, hospitalization, the number of relevant studies is limited.

Among NHIC beneficiaries from 0 to 5 years of age in the present study, the total number of patients increased by 62.7% and the total number of cases increased by 66.6% after compared with before policy implementation. After policy implementation, the number of inpatient days per case decreased by 6.4% and the hospital expenditure per case decreased by 9.1%. Among the NHIC beneficiaries, the magnitude of change was bigger in industrial worker group than in Self-employed. Therefore, the intensity of health care utilization decreased with an increase in total health care utilization. This may have resulted from an increase in medical utilization, which in turn caused a decrease in the intensity of health care utilization [13]. The trend of medical aid beneficiaries who were already exempt from the copayment waiver policy showed similar results during the same period. The net effect of the policy on this finding is unclear because the trend of health care intensity decreased. A comparison of NHIC beneficiaries and medical aid beneficiaries was conducted to estimate the net effect. In the regression analysis using the “difference in difference”, the intensity of medical utilization did not remain consistent. With respect to the number of inpatient days per case, the NHIC beneficiaries displayed less negative results than did the medical aid beneficiaries. In other words, the effect of the policy actually increased the number of inpatient days per case. With respect to the hospital expenditure per case, that of the NHIC beneficiaries group decreased by more than did that of the medical aid beneficiaries group, and the policy did not increase the hospital expenditure per case. Therefore, it cannot be stated that the copayment waiver policy changed the intensity of medical utilization [21]. Other studies about copayment implementation also changed the treatment pattern and but overall effect on cost is mixed. These results imply that the policy on copayment could result in unintended effects due to the response of patients and supplier, and should be implemented with considerable caution [16,17].

The interactions between time and the stated variables were considered in the analysis of the effects of disease and income level on policy use among NHIC beneficiaries. According to the regression analysis, patients with fatal diseases acted differently than did those with non-fatal diseases. The intensity of healthcare utilization increased after implementation of the copayment waiver policy in terms of both for the number of inpatient days per case and the hospital expenditure per case. Because the copayment waiver policy aimed to cover catastrophic events and hospital expenditure, the increase in the intensity of health care utilization was appropriately targeted by the policy. In terms of the income level, the intensity of medical use among the low-, low-middle, and middle-income groups was negatively affected by the copayment waiver policy compared with the high-income group. Because low-income groups are known to be sensitive to cost sharing, this was contradictory to the results of previous studies [13,14,21]. The deviation from our expectations may have been due to the inclusion of hospitalized children in this study.

Before the policy implementation, the intensity of health care use for NHIC beneficiaries was anticipated. Also among NHIC beneficiaries, the patients who had a fatal disease and were low income class were major target of the policy. But our result show that though the fatal patients had higher intensity, the intensity of high income class increased more. Also when compared with medical aid group, the net effect of policy among NHIC beneficiaries was unclear. Therefore, it is hard to say the purpose of policy was accomplished.

Several limitations must be considered when interpreting the results of this study. First, the effect of cost on hospital care and length of stay is limited [22]. Therefore, the effect of copayment waiver policy is also restricted. In addition, when using the difference in difference method, the intervention events must be exempt, and other characteristics should be similar between the groups to obtain an exact estimation. In this study, the medical aid beneficiaries group was poorer and less healthy than was the NHIC beneficiaries group, and the medical needs of the two groups were different [23]. Therefore, the estimate of policy effect could be biased. Also the characteristics of parents except income were not considered. Additionally, the accuracy of assessing the income of self-employed individuals in Korea has been questioned. Because the premium level was based on the assessed income, our analysis might have been affected by the inaccuracy of income assessment.

## Conclusions

This study involved a natural experiment involving the copayment waiver policy. The co-payment waiver policy did not decrease the intensity of health care utilization compared with that of the medical aid beneficiaries group. Furthermore, the intensity of medical use increased in the fatal disease group, but did not improve in the low-income group. Our studies have important policy implications in Korea and other countries in terms of the level of patient cost-sharing of medical costs.
